# Prevalence of Acanthosis Nigricans in an urban population in Sri Lanka and its utility to detect metabolic syndrome

**DOI:** 10.1186/1756-0500-4-25

**Published:** 2011-01-28

**Authors:** Anuradha S Dassanayake, Anuradhani Kasturiratne, Madunil A Niriella, Udaya Kalubovila, Shaman Rajindrajith, Arjuna P de Silva, Norihiro Kato, A Rajitha Wickremasinghe, H Janaka de Silva

**Affiliations:** 1 Faculty of Medicine, University of Keleniya, Ragama, Sri Lanka; 2 International Medical Centre of Japan, Tokyo, Japan

## Abstract

**Background:**

Insulin resistance (IR) plays a major role in the pathogenesis of metabolic syndrome. Acanthosis nigricans (AN) is an easily detectable skin condition that is strongly associated with IR. The aims of this study were, firstly, to investigate the prevalence of AN among adults in an urban Sri Lankan community and secondly, to describe its utility to detect metabolic syndrome.

**Findings:**

In a community based investigation, 35-64 year adults who were selected using stratified random sampling, underwent interview, clinical examination, liver ultrasound scanning, and biochemical and serological tests. Metabolic syndrome was diagnosed on revised ATP III criteria for Asian populations. AN was identified by the presence of dark, thick, velvety skin in the neck.

2957 subjects were included in this analysis. The prevalence of AN, metabolic syndrome and type 2 diabetes mellitus were 17.4%, 34.8% and 19.6%, respectively. There was a strong association between AN and metabolic syndrome. The sensitivity, specificity, positive predictive value and negative predictive value of AN to detect metabolic syndrome were 28.2%, 89.0%, 45.9% and 79.0% for males, and 29.2%, 88.4%, 65.6% and 62.3% for females, respectively.

**Conclusions:**

AN was common in our study population, and although it did not have a high enough sensitivity to be utilized as a screening test for metabolic syndrome, the presence of AN strongly predicts metabolic syndrome.

## Background

Acanthosis nigricans (AN) is an easily identifiable skin lesion characterized by velvety, brownish-black pigmentation of the skin of the neck and intertriginous surfaces. Typical areas of involvement include the posterior aspect of the neck, axillae, elbows and knees; the neck is involved 93% to 99% of the time [1,2]. The common occurrence of AN in the neck is important in a primary care setting because it makes the lesion easily detectable. A quantitative scale of AN has been developed by Burke et al [1]. This scale takes into consideration the severity of AN in neck and axilla, neck texture, and the presence or absence of AN in knuckles, elbows and knees.

A number of studies have shown an association between AN and insulin resistance [3,4] and the recent increase in the prevalence of AN [5-7] may well reflect increasing trends in obesity and type 2 diabetes worldwide [1]. The prevalence of AN varies from 7% in unselected populations to 74% in obese people [5,6]. It has been shown to be a reliable cutaneous marker of insulin resistance in obese Japanese children [8]. The prevalence also varies in different racial groups. For example, African Americans are 25 times more likely to have AN than patients of European descent [2]. A study from the USA reports that the prevalence of AN was 3% among Caucasians, 19% in Hispanics and 28% in American Indians [7]. Although obesity increases the risk of AN, racial differences in the prevalence of AN cannot solely be explained by different rates of obesity. Although a hospital based study from India has reported a prevalence of AN of 65% among diabetics and 40% among healthy people attending non-diabetic patients [9], to our knowledge there are no data on community prevalence of AN from the Indian subcontinent.

The diagnosis of metabolic syndrome involves a battery of investigations that poses a challenge to doctors working in poor countries, particularly in a primary care setting. In these situations, where there are many competing demands within the brief patient encounter, a clinical marker to rapidly identify persons at high risk for metabolic syndrome would be very useful. AN may be a suitable clinical marker especially in communities where it is prevalent.

The aims of this study were, firstly to investigate the prevalence of AN in an urban Sri Lankan community, and secondly, to describe its utility to detect metabolic syndrome.

## Materials and methods

**Figure 1:**
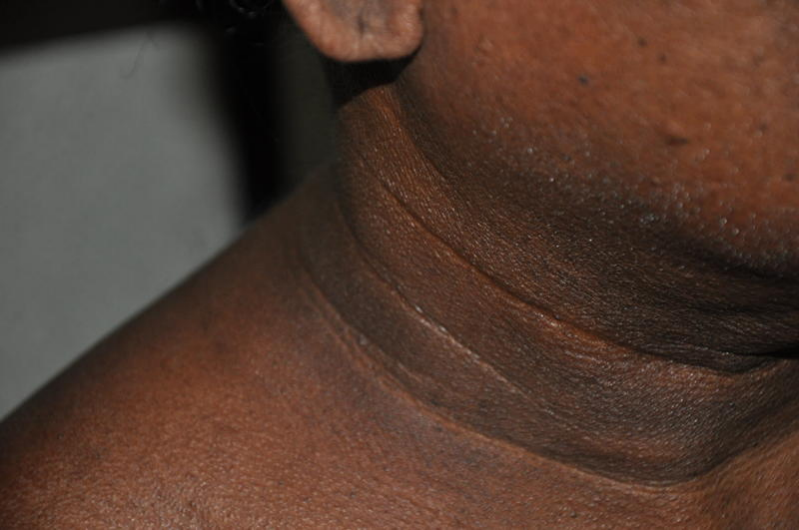
**A patient with acanthosis nigricans from the study**.

This study was part of a community based investigation - the Ragama Health Study (RHS), conducted in the Ragama Medical Officer of Health area [10]. This area has characteristics typical of an urban community in Sri Lanka. Participants were a representative sample of 35-64 year old adults selected by stratified random sampling from electoral lists. Ethical approval for the study was obtained from the Ethics Review Committee of the Faculty of Medicine University of Kelaniya. The purpose of the study, the procedures to be carried out with potential hazards and benefits were explained to the individuals prior to obtaining informed written consent. Consenting adults were screened by a structured interview, clinical examination, liver ultrasound (8 MHz probe, Toshiba ultrasound diagnostic systems SSA-51 OA, Toshiba Medical Systems Corporation, Otawara-city, Tochigi-prefecture, Japan) and collection of 10 ml venous blood for biochemical tests. Metabolic syndrome was diagnosed on revised ATP III criteria for Asian populations [11]. AN was identified by the presence of dark, thick, velvety, pigmented skin in the neck (Figure 1). The person appearing in the photograph (Mr KDEPN) has given informed written consent for it to be published.

### Statistical analysis

Data were entered in Epi Info 2000 (Centres for Disease Control and Prevention, Atlanta. GA) and logical and random checks were done. Statistical analysis was done using SPSS version 16.0 (SPSS Inc., Chicago, IL). Continuous and categorical data were described using mean and standard deviations. Significance testing was done using the Student T test, Chi squared test and multiple logistic regression. p < 0.05 was considered as significant. The sensitivity, specificity, positive predictive value (PPV), and negative predictive value (NPV) of AN to detect metabolic syndrome were calculated.

## Results

3012 subjects participated in the study and 2957 had information on the presence or absence of AN (Table 1, 2). 515 (17.4%) had AN, and 1025 (34.8%) had metabolic syndrome diagnosed by revised ATP III criteria (Table 3, 4). Prevalence of type 2 diabetes mellitus was 18.9% among males and 20.1% among females giving an overall prevalence of 19.6% (n = 584). There were 692 females (67.5%) with metabolic syndrome (OR = 2.29; 95% CI: 1.96-2.69). Age distribution between sexes was similar [males: mean 52.4 years (SD = 7.9) vs. females: mean 52.4 years (SD 7.8)]. AN was more common among both males [28.2% vs. 11.0% (OR = 3.18; 95% CI: 2.34-4.34)]; and females [29.2% vs. 11.6% (OR = 3.15; 95% CI: 2.43-4.08)} with metabolic syndrome. The sensitivity, specificity, PPV and NPV of AN to detect metabolic syndrome were 28.2%, 89.0%, 45.9%, and 79.0% for males, and 29.2%, 88.4%, 65.6% and 62.3% for females, respectively.

**Table 1:** Demographic and anthropometric variables of subjects with and without AN

Variable	Subjects with AN	Subjects without AN	p value
Males [Number (%)]	206 (40)	1138 (47)	0.006
Age [Mean (SD)]	50.8 (7.6)	52.8 (7.8)	< 0.001
BMI [Mean (SD)]	27.9 (4.0)	23.3 (3.9)	< 0.001
Waist [Mean (SD)]	93.9 (9.1)	83.9 (10.2)	< 0.001
Hip [Mean (SD)]	97.5 (8.3)	89.9 (8.0)	< 0.001
WHR	0.96 (0.07)	0.93 (0.07)	< 0.001

**Table 2:** Demographic and anthropometric factors in subjects with and without metabolic syndrome and acanthosis nigricans

Variable	Acanthosis nigricans (subjects with metabolic syndrome n = 1025)			Acanthosis nigricans (subjects without metabolic syndrome n = 1924)		
	**Present**	**Absent**	***p *value**	**Present**	**Absent**	***p *value**
	**n = 296 (28.9)**	**n = 729 (71.1)**		**n = 217 (11.3)**	**n = 1707 (88.7)**	

Age	51.8 (7.4)	54.6 (7.0)	< 0.001	49.4 (7.8)	52.0 (8.0)	< 0.001

Males	94 (31.8)	239 (33.0)	0.71	111 (51.2)	899 (52.5)	0.72

BMI	28.4 (4.0)	25.5 (3.4)	< 0.001	27.2 (3.8)	22.4 (3.8)	< 0.001

Waist	95.3 (8.5)	90.3 (8.6)	< 0.001	92.0 (9.4)	81.2 (9.6)	< 0.001

WHR	0.97 (0.07)	0.96 (0.07)	< 0.001	0.96 (0.07)	0.92 (0.07)	0.02

**Table 3:** Association of Acanthosis Nigricans with revised ATP III criteria for diagnosis of metabolic syndrome in Asians (based on bivariate analysis)

Criterion	Odds Ratio	95% Confidence limits	P value
Abdominal obesity	5.64	4.41-7.22	< 0.001
(Waist circumference) Males > 90 cm			
Females > 80 cm			

Triglycerides ≥150 mg/dl	1.81	1.49-2.20	< 0.001

High-density lipoprotein cholesterol	1.56	1.25-1.94	< 0.001
Males < 40 mg/dl			
Females < 50 mg/dl			

Blood pressure ≥130/≥85 mm Hg	2.04	1.66-2.51	< 0.001

Fasting glucose > 110 mg/dl	1.85	1.53-2.25	< 0.001

**Table 4:** Association of Acanthosis Nigricans with revised ATP III criteria for diagnosis of metabolic syndrome in Asians (based on multiple logistic regression)

Criterion	β	Odds Ratio Exp (β)	95% Confidence limits	P value
Abdominal obesity	1.553	4.72	3.67-6.07	< 0.001
(Waist circumference)				
Males > 90 cm				
Females > 80 cm				

Triglycerides ≥150 mg/dl	0.347'	1.42	1.15-1.75	0.001

High-density lipoprotein cholesterol	0.274	1.32	1.05-1.66	0.020
Males < 40 mg/dl				
Females < 50 mg/dl				

Blood pressure ≥130/≥85 mm Hg	0.394	1.48	1.19-1.85	0.001

Fasting glucose > 110 mg/dl	0.356	1.43	1.16-1.75	0.001

## Discussion

The prevalence of AN in this urban adult population from Sri Lanka was a relatively high 17.4%. To our knowledge, this is the first community based prevalence study of AN from the Indian subcontinent. Our results are comparable with prevalence data in American Indians, African Americans and Hispanics. As expected, we found that AN was significantly more common in people with metabolic syndrome. We also found that AN had a high specificity and NPV, but a low sensitivity to detect metabolic syndrome. One of the shortcomings in our study was that we did not quantify AN using a standard scale, and only its presence or absence in the neck was noted. However, we feel that if AN is to be a useful clinical marker in primary care, its detection would be most likely in an exposed area of skin.

Patients with AN are at risk for all components of the metabolic syndrome, such as, obesity, hypertension, elevated triglycerides, low HDL, and impaired glucose tolerance [12,13]. AN is also highly prevalent in certain ethnic groups [5], and in such populations the easy detectability increases its potential to play a bigger role in detecting people at risk of metabolic syndrome. This would be relevant to many developing countries with poor resources, such as those in South Asia, which are battling rapid increases in diabetes, obesity and other components of the metabolic syndrome. Despite this, there are few studies investigating the role of AN as a clinical marker to detect people who are at risk of having metabolic syndrome. In a recent study in the USA, 49% of 676 fifth grade children with AN fulfilled criteria for metabolic syndrome [14]. Some states in the USA have encouraged their doctors to look for AN in children in order to detect those at high risk of developing diabetes mellitus [15]. Several recent studies recommend the use of AN as a marker of insulin resistance in American Indian children [16].

Although AN was common in our study population, its low sensitivity does not make it a suitable screening test to detect metabolic syndrome. However, the high specificity and NPV make AN a very useful sign to predict its presence. Primary health care workers and doctors in resource poor settings should be trained to look for and identify AN, and to refer those with the lesion for further investigation and assessment of insulin resistance and metabolic syndrome.

## Competing interests

The authors declare that they have no competing interests.

## Authors' contributions

HJdeS, ARW, NK conceptualized and designed the study. ASD, APdeS, SR, UK, MAN and AK acquired, analyzed and interpreted the data. MAN, ASD and AK drafted the initial version of the manuscript. All authors critically reviewed and revised the manuscript and contributed to the preparation of the final draft. All authors approved the final version of the manuscript.
